# Solid-State NMR Investigation of Electrolyte Effects on Silicon–Graphite Composite Anode: Solid Electrolyte Interphase Formation and Failure Mechanisms

**DOI:** 10.1021/acs.chemmater.5c02476

**Published:** 2026-04-23

**Authors:** Nahom Enkubahri Asres, Marta Cabello, Muhammad Khurram Tufail, Kerman Gomez Castresana, Aitor Villaverde, Juan Miguel López del Amo

**Affiliations:** † Centre for Cooperative Research on Alternative Energies (CIC energiGUNE), Basque Research and Technology Alliance (BRTA), Alava Technology Park, Albert Einstein 48, Vitoria-Gasteiz 01510, Spain; ‡ Departament of Organic and Inorganic Chemistry, Faculty of Science and Technology, University of the Basque Country, UPV/EHU, B° Sarriena s/n, 48940 Leioa, Spain; § Division 6.3 Structure Analysis, Bundesanstalt für Materialforschungund-Prüfung (BAM), Unter den Eichen 87, 12203 Berlin, Germany

## Abstract

Silicon (Si) is a promising anode material due to its high specific capacity (∼3580 mAh g^–1^), far exceeding that of graphite (∼372 mAh g^–1^). However, its large volumetric expansion (∼300%) during lithiation induces mechanical stress, fracturing particles, and repeatedly exposing fresh surfaces to the electrolyte. This leads to continuous SEI growth, consuming lithium and electrolyte, and causing rapid capacity fading. To address these issues, strategies such as incorporating Si into graphite (Gr) composites and optimizing electrolytes have shown promise in improving the stability and performance of Si-based anodes. NMR spectroscopy offers element-specific sensitivity and can probe local chemical environments, making it a powerful tool for examining both the surface and bulk properties of battery materials. In this work, we use solid-state NMR spectroscopy to investigate Si/Gr anodes in two systematically chosen electrolytes: one EC-based (known to form organic-rich SEI) and one FEC-based (inorganic-rich SEI). We conducted 1D ^7^Li, ^19^F, and ^1^H NMR experiments to elucidate the lithiation mechanism and identify SEI components in Si/Gr composite anodes during the first cycle and after extended cycling in the fully lithiated state for these two electrolyte systems. Additionally, we performed cross-polarization (CP) and two-dimensional exchange spectroscopy (EXSY) NMR experiments to gain deeper insight into Li^+^ coordination within different SEI components and to probe dynamic exchange processes between the SEI and lithiated Si/Gr phases (Li_*x*_Si/Li_*x*_C_6_). ^1^H/^19^F → ^7^Li CP-MAS EXSY NMR was employed to selectively probe Li^+^ exchange originating from either the organic or inorganic fraction of the SEI. These NMR results were correlated to the electrochemical performance of the Si/Gr anode in both electrolyte systems.

## Graphical abstract

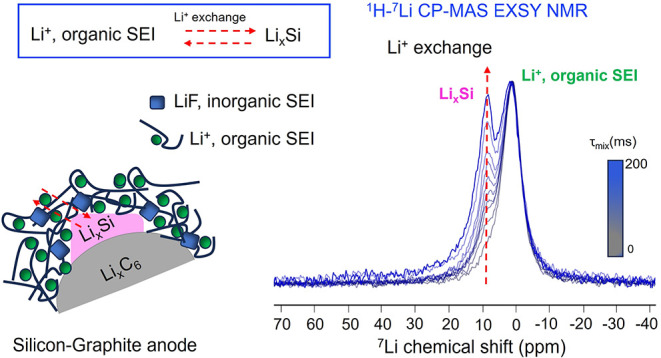

## Introduction

Silicon (Si) became a promising anode material due to its exceptionally high theoretical capacity (∼3580 mAh g^–1^ in the fully lithiated state, Li_3.75_ Si), which is nearly an order of magnitude greater than that of conventional graphite (Gr) anodes (∼372 mAh g^–1^).^1–3^ Moreover, the low discharge potential of Si (∼400 mV) makes it well suited for high-energy-density batteries.^4^ Despite these advantages, the practical implementation of Si anodes is hindered by significant volumetric changes (>300%) during lithiation and delithiation, which induce substantial mechanical stress.^5,6^ This stress often causes the pulverization of Si particles and their isolation from both the conductive matrix and the current collector, compromising the anode’s structural integrity and disrupting the electrical pathways essential for efficient battery performance.^7–10^

These cracking or pulverization of Si particles, both in bulk and at the surface, expose fresh silicon to the electrolyte, triggering continuous formation and growth of the solid-electrolyte interphase (SEI). This accumulating SEI increases cell resistance and leads to incomplete reactions, reducing the utilization of active material and generating electronically isolated species (inactive Li^+^ or Li_*x*_Si), either temporarily or permanently.^11–13^ Excess formation of Li^+^-containing SEI species consumes limited active Li-ions from the reservoir in a full cell, reducing the reversible capacity during cycling.^14^ To address these challenges, various methodologies have been proposed, such as conductive coatings,^15,16^ reduction of Si particle size,^17,18^ and modifications of the electrolyte composition.^19,20^

One of the most promising strategies involves integrating Si particles into graphite, leading to the development of Si/Gr composite anodes.^15,21^ These anodes combine the mechanical stability and electrical conductivity of graphite with the high capacity of silicon.^22,23^ However, despite these advantages, Si/Gr composite anodes still experience capacity fading over extended cycling when they are used with conventional EC-based electrolytes. This is largely attributable to the significant volume changes that induce mechanical stress on the SEI, causing cracks and re-exposing Si particles to the electrolyte, which leads to uncontrolled electrolyte decomposition and hinders stable SEI formation.^24–26^ An alternative approach to mitigate these issues is the use of electrolyte additives such as fluoroethylene carbonate (FEC), which is known to stabilize the SEI, therefore reducing capacity loss and improving cycling stability.^28–30^

Although Si-containing anodes and their failure mechanisms have been extensively investigated using various spectroscopic techniques and electrochemical methods, further improvements in these systems are still emerging.^25,27–37^ However, a significant gap remains in understanding the composition, structure, and dynamics of the electrolyte decomposition product in the SEI, which influence Li-ion transport across the interphase. Correlating these SEI decomposition products and their evolution with the failure mechanisms of Si anode-based systems—whether on the surface or in the bulk—during extended cycling is crucial for designing a more compatible electrolyte-electrode system with superior electrochemical performance.

In this work, we apply multinuclear and multidimensional solid-state NMR techniques to investigate both the surface decomposition products and bulk properties of Si/Gr anodes using two systematically selected electrolytes: one based on an ethylene carbonate (EC) solvent and the other based on a fluoroethylene carbonate (FEC) solvent. Although previous studies have used solid-state NMR to examine Si anodes—either focusing on SEI decomposition products, which revealed various organic and inorganic species within the SEI,^38–40^ or on reaction mechanisms that identify different lithium silicides (Li_*x*_Si) formed electrochemically or chemically, ^12,41–48^ there remains a gap in correlating the formed SEI with the failure mechanisms occurring in the bulk of Si particles. Therefore, we employed solid-state MAS NMR to investigate the effects of two electrolytes (EC-based and FEC-based) on cycled Si/Gr composite electrodes both at early stage cycling and after extended cycling. This approach allows us to identify the composition of the SEI on the electrode surface and its evolution as well as track the distribution of Li-ions in the bulk electrode—within both graphite and Si, over multiple cycles. In addition to one-dimensional multinuclear direct-excitation measurements, we utilized various solid-state NMR techniques, such as cross-polarization (CP) to differentiate Li-ions embedded to the organic and inorganic components, and exchange spectroscopy (EXSY) to examine Li-ion exchange between lithiated silicon (Li_*x*_Si) or lithiated graphite (Li_*x*_C_6_) and the SEI.

In this work, we employed an innovative CP-EXSY NMR approach to selectively probe Li-ion exchange between the SEI and lithium in the bulk electrode, determining whether it originates from the organic or inorganic phases of the SEI (or both). This method also revealed that the majority of the exchange proceeds through the organic component of the SEI in both electrolyte systems (EC- and FEC-based) and enabled us to investigate both the extent (fraction) and the rate of this exchange process. Furthermore, our NMR results, together with comprehensive electrochemical analysis allowed us to identify the cause of capacity fading during the extended cycling when using EC-based electrolytes.

## Experimental Section

### Materials and Electrochemistry

For the preparation of silicon-graphite (Si/Gr) composite, the ball milling approach was followed as previously reported by our group.^49^ Briefly, silicon nanoparticles (Alfa Aesar) were mixed with graphite (SFG15L, Imerys) in a weight ratio of 37.5:62.5 in a planetary mill (Pulverisette, Fritsch) at 400 rpm for 2 h with pauses for cooling. 10 mL of isopropyl alcohol (IPA) (Scharlab) were added to achieve better dispersion and distribution of the particles. For electrode fabrication, the Si/Gr composite active material was mixed with lab-made lithium polyacrylate (LiPAA) binder and C45 conductive additive, according to the formulation 85:7.5:7.5 (AM/binder/conductive additive), in a dissolver (Dispermat CV3-PLUS) with a total processing time of 4 h and a maximum speed of 1200 rpm. The slurry was then cast onto copper foil by using a Dr Blade and dried. The resulting electrodes had an area loading of 2.0 mAh/cm^2^, with 31 wt % of silicon at the electrode level.

The electrochemical measurements were performed in CR2032-type coin cells assembled inside a glovebox under an argon atmosphere. The half cells were assembled using Si/Gr as a positive electrode, a disc of metallic lithium as the negative one, and a Whatman glass fiber disc as a separator of both electrodes. As for the electrolyte, two different formulations were tested: (i) 1 M lithium hexafluorophosphate (LiPF_6_) in fluoroethylene carbonate (FEC) and ethyl methyl carbonate (EMC) (FEC:EMC = 3:7 wt %) plus 2 wt % vinylene carbonate (VC) (e-lyte), and (ii) 1 M LiPF_6_ in ethylene carbonate (EC) and EMC (EC:EMC = 3:7 wt %) (e-lyte). In this work, the former electrolyte will be referred to as “FEC-based,” and the latter as “EC-based”. Galvanostatic experiments were run in a MACCOR battery tester in the 0.01–0.90 V voltage window with a current rate of C/15. The value of C was determined using an estimated capacity of 1575 mAh g^–1^ (based on 37.5% Si with a specific capacity of 3579 mAh g^–1^, and 62.5% graphite with a specific capacity of 372 mAh g^–1^).

### Solid-State NMR Measurements

Si/Gr anodes for solid-state NMR measurements were electrochemically cycled in half cells against lithium metal at C/15 from the open-circuit voltage (OCV) to 10 mV. After the first lithiation cycle, the electrodes were extracted from the cells and rinsed with dimethyl carbonate (DMC) inside a glovebox. The lithiated silicon–graphite electrodes were then scraped from the copper current collector, mixed with KBr to facilitate sample spinning, and packed into 2.5 mm rotors. Two electrodes (each comprising a 12 mm diameter disc) were used to fill each rotor for both the EC- and FEC-based systems.

Magic Angle Spinning Nuclear Magnetic Resonance (MAS NMR) was performed by using a Bruker Avance III 500 MHz (*B*_0_ = 11.7 T) spectrometer. Samples were measured using 2.5 mm rotors and with a spinning frequency of 20 kHz. ^6^Li and ^7^Li chemical shifts were referenced to a 0.1 M aqueous LiCl solution (δ = 0 ppm), calibrated using the bulk-water ^1^H signal at 4.7 ppm. ^19^F chemical shifts were referenced to solid LiF (δ = −204 ppm). A rotor-synchronized Hahn echo pulse sequence was used with a recycle delay that was at least 5 times *T*_1_ to allow for quantitative analysis. The 1D ^19^F and ^1^H NMR spectra were recorded using the Hahn echo pulse sequence with the 90° pulse lengths of ∼3 μs and ∼2.1 μs for ^19^F and ^1^H, respectively. 2D ^7^Li–^7^Li EXSY and ^6^Li–^6^Li EXSY measurements were performed using a 90°–*t*_1_–90°−τ_mix_–90°–*t*_2_ pulse sequence with mixing times (τ_mix_) of 100 and 200 ms, respectively, which provided the best compromise between diagonal and off-diagonal signal intensities. All 2D spectra were recorded with 256 scans for each of the 80 *t*_1_ increments. The recycle delay was set to 2 s for the ^7^Li–^7^Li EXSY experiments and 10 s for the ^6^Li–^6^Li EXSY experiments. The 90° pulse length was 2.4 μs. ^19^F → ^7^Li CP-MAS experiments were conducted with a contact time of 600 μs, a recycle delay of 5 s, and a time between 1024 and 2048 scans. ^1^H → ^7^Li CP-MAS experiments were performed with a contact time of 1000 μs; for each sample, 10240 scans were acquired by using a recycle delay of 3 s.

For the ^1^H/^19^F → ^7^Li CP-MAS EXSY experiments, a modified EXSY sequence employing ^1^H → ^7^Li or ^19^F → ^7^Li cross-polarization (CP-MAS EXSY) was used instead of single-pulse excitation. The experiments were conducted at a spinning frequency of ω/2π = 20 kHz, where ω is the angular frequency. For the ^1^H → ^7^Li CP-MAS EXSY experiment, a recycle delay of 2 s and a cross-polarization contact time of 1300 μs were used. For the ^19^F → ^7^Li CP-MAS EXSY experiment, the recycle delay was 10 s and the contact time was 600 μs. In both cases, the mixing time (exchange period) varied from 1 ms to 200 ms. ^19^F–^7^Li Heteronuclear correlation (HETCOR) experiments were recorded by applying a cross-polarization (CP) ^19^F–^7^Li magnetization transfer step of 800 us. Rotor temperature calibration was carried out at different MAS frequencies using lead nitrate as a temperature calibration standard.^50^ Based on these measurements, spinning at 20 kHz (2.5 mm rotor), as used in this work, results in an additional heating of 17.4 °C, leading to an actual sample temperature of approximately 39 °C.

## Results

### Electrochemistry: Galvanostatic Cycling

Galvanostatic cycling of the Si/Gr composite was conducted separately by using each of the two electrolyte systems (FEC- and EC-based) to identify the different electrochemical processes. In Figure 1a, the voltage profiles of Si/Gr for two consecutive discharge/charge cycles are presented. Overall, the voltage-capacity profiles are very similar for both electrolyte systems containing cells, indicating that FEC- and EC-based electrolytes do not significantly affect the initial lithiation and delithiation mechanisms of the Si/Gr anode. During the first lithiation (formation cycle), both cells exhibit a relatively steep drop from open circuit voltage to ∼250 mV corresponding to SEI formation with a capacity of 200–250 mAh g^–1^. This capacity is attributed to the reduction of FEC and EC, as both Si and Gr have lithiation potentials below 250 mV. However, the irreversible capacities for FEC and EC are 480 and 440 mAh g^–1^, respectively, indicating that the SEI may continue to form below 250 mV or that, possibly, there is Li^+^ trapping in the form of an amorphous silicide (a-Li_*x*_Si). A minor difference between the FEC- and EC-based electrolyte cells appears in the SEI-formation potential range: the FEC-based electrolyte shows a minor plateau around 1.3 V vs Li/Li^+^, corresponding to FEC decomposition, whereas the EC-based electrolyte predominantly decomposes around 600 mV. After SEI formation, both electrolyte systems exhibit a short plateau at approximately 200 mV, corresponding to the onset of Li-ion intercalation into graphite. It is well-known that the first lithiation of crystalline silicon (c-Si) follows a two-phase reaction, wherein crystalline Si (c-Si) transitions to amorphous lithium silicide (a-Li_*x*_Si). Consistent with previous reports,^1,51^ this initial lithiation occurs ∼100 mV, as seen in the voltage profile as a flat plateau (Figure 1a).

**Figure 1:**
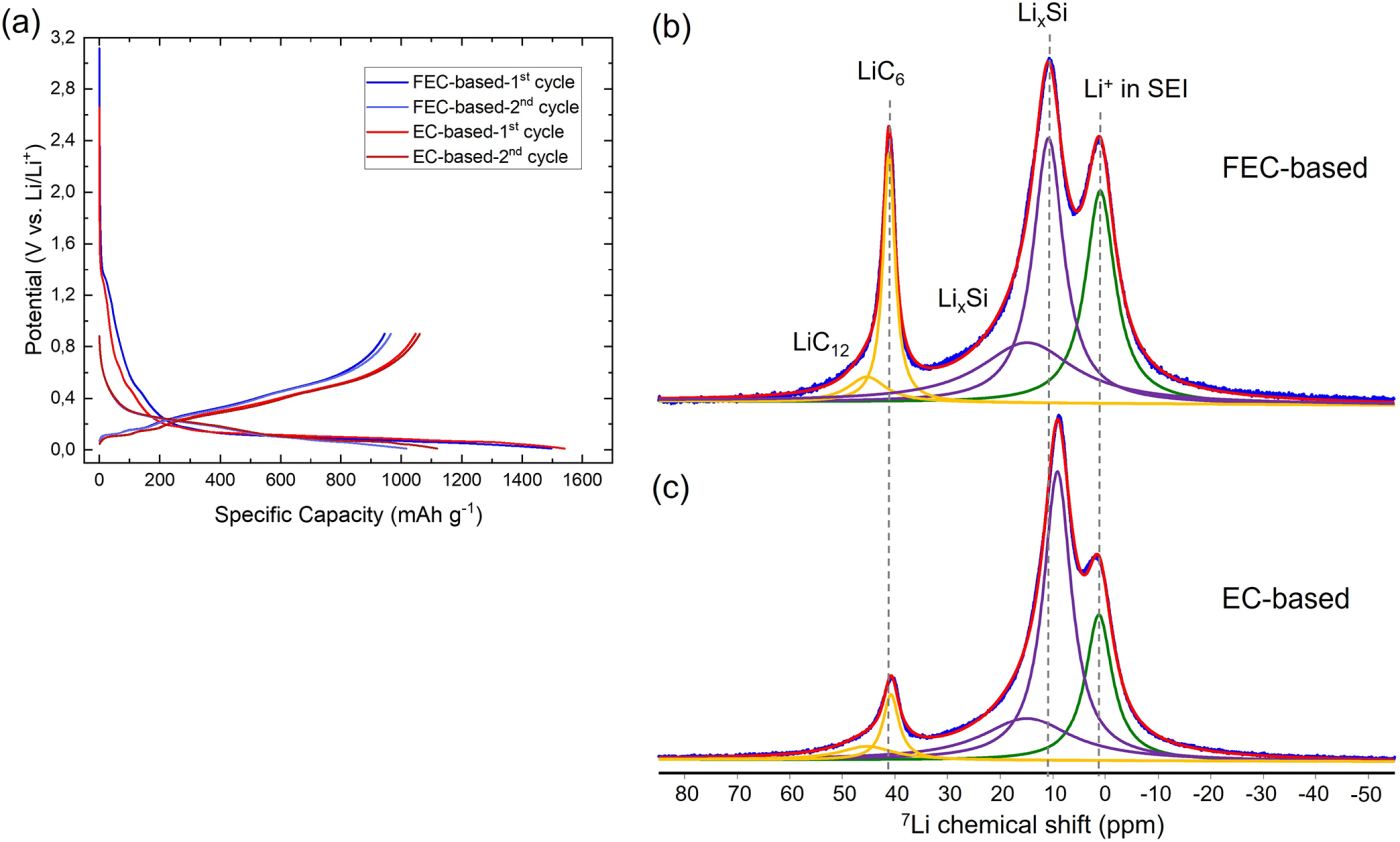
(a) Voltage profiles of Si/Gr anodes cycled in cells with EC-based (red) and FEC-based (blue) electrolytes at a C-rate of C/15. ^7^Li solid-state NMR spectrum of the ex-situ Si/Gr anode recovered after the first lithiation in the (b) FEC-based electrolyte and (c) EC-based electrolyte. The lithiation cutoff voltage was set to 10 mV.

While the lithiation features of silicon and graphite are largely superimposed during the first lithiation at potentials below 100 mV vs Li/Li^+^, the first charge (delithiation) shows two short plateaus at approximately 110 and 150 mV. These plateaus, which are not present for pure Si,^1^ are attributed to the delithiation of graphite. Specifically, the plateau at ∼110 mV corresponds to the transition from LiC_6_ to LiC_12_, whereas the plateau at ∼150 mV corresponds to the transition from LiC_12_ to LiC_18_ (dilute stages 2 and 3) consistent with previous results.^52^ Following the delithiation of graphite, the potential profile exhibits two pseudoplateaus at approximately 250–400 and 400–550 mV, characteristic of the delithiation of amorphous Li_*x*_Si (Li_*x*_Si → a-Si + xLi^+^), specifically to the formation of phases with approximate stoichiometries a-Li_∼2_Si (a-Li_∼3.5–3.75_Si → a-Li_∼2.0_Si) and a-Si (a-Li_∼2.0_Si → a-Si), respectively.^44–46^ During the second lithiation, the mechanism differs from the first lithiation, as observed from the potential profiles. In this cycle, first lithiation of amorphous Si was evidenced by the sloping potential profile between 300–180 and 180–80 mV, corresponding to the lithiation stages with a stoichiometry ratio of a-Li_∼2_Si (a-Si → a-Li_∼2.0_Si) and a-Li_∼3.5–3.75_Si (a-Li_∼2.0_Si → a-Li_∼3.5–3.75_Si), respectively. During the second delithiation, a potential profile similar to that of the first delithiation is observed, suggesting that once c-Si is amorphized (a-Li_*x*_Si) during the initial lithiation, the reaction mechanism of Li_*x*_Si becomes symmetric in subsequent cycles.

### Solid-State NMR Studies: Ex-Situ NMR on First Cycle Lithiated Si/Gr Anodes

Ex-situ ^7^Li MAS NMR characterization was performed on Si/Gr anode materials recovered after the first lithiation (with a lower cutoff potential of 10 mV) from cells cycled in EC- and FEC-based electrolytes, as shown in Figure S1. As already reported in previous research, NMR can very well distinguish between lithium in different crystalline and amorphous lithium silicide (Li_*x*_Si) phases as well as in graphite in the different graphite intercalation compound (GIC) stages.^42–45,47,48,53,54^ In agreement with these works, the resulting ^7^Li NMR spectra were assigned for both FEC- (Figure 1b) and EC-based (Figure 1c) cycled Si/Gr anodes revealing three primary resonances with distinct chemical shift at ∼42, ∼10, and ∼0 ppm, corresponding to lithiated graphite, Li_*x*_Si silicide phases formed during silicon lithiation, and diamagnetic Li^+^ ions at the SEI, respectively.

Deconvolution of the ^7^Li NMR spectra (Figure 1b,c) shows that both electrolyte systems display NMR signals at similar chemical shifts for the corresponding lithiated graphite resonances centered around 42 ppm. These signals were deconvoluted considering two components for both electrolyte system: the dominant LiC_6_ corresponding to dense lithiation stage 1 (majority signal at ∼41 ppm) and a minor component of LiC_12_ corresponding to dense lithiation stage 2 (∼45 ppm) in agreement with the assignments made in previous works.^53,54^ However, a slight chemical shift difference was observed for the lithiated silicon signals (Li_*x*_Si): ∼11 ppm for the FEC-based electrolyte and ∼9 ppm for the EC-based electrolyte. According to previous studies by Key et al.^42,43^ and Ogata et al.,^44^ the signals appearing around the observed chemical shifts in our work were assigned to Li-ions located near both small Si–Si clusters and isolated Si ions in similar environments as found in the model compound Li_13_Si_4_ and a-Li_3.5–3.75_ Si, although predominantly associated with the latter. Overall, lower chemical shifts correspond to highly lithiated (Li-rich) amorphous silicon phases.^41–44^ The observed spectra also reveal the presence of the amorphous a-Li_3.5–3.75_Si phase, as indicated by the resonances observed at approximately 11 and 9 ppm for the FEC- and EC-based electrolyte systems, respectively. This is consistent with the electrochemically measured capacities at a lower cutoff potential of 10 mV, as shown in Figure S1.

Moreover, broad diamagnetic ^7^Li NMR signals, centered at ∼0 ppm, are observed in the spectra of both EC- and FEC-based spectra presented in Figure 1b,c. The small differences can be attributed to distinct Li^+^ coordination or local environments expected by the different chemical compositions of the EC- and FEC-based SEI formed. Due to the presence of multiple organic and inorganic Li-containing species, it is not possible to resolve the rather broad signal at ∼0 ppm for these components individually using only the ^7^Li NMR spectra obtained by direct excitation. However, the element-selective nature of NMR can still be leveraged to identify SEI decomposition products. To further analyze these products, 1D ^19^F and ^1^H NMR experiments were performed on both samples, (Figure 2a,b).

**Figure 2:**
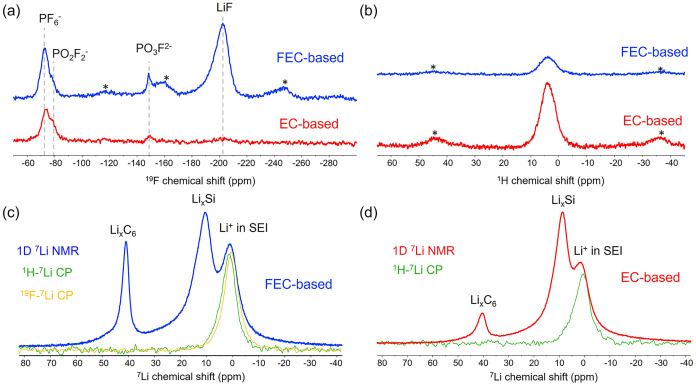
^19^F and ^1^H NMR spectra obtained from the ex-situ Si/Gr anode of the EC- and FEC-based electrolyte samples discharged to 10 mV are shown in (a) and (b). The corresponding ^1^H → ^7^Li and ^19^F → ^7^Li CP-MAS NMR spectra are shown in (c) and (d) superposed with the original 1D ^7^Li NMR spectra. MAS rotational sidebands are denoted by asterisks (*). Note: The spectra are scaled/normalized by the amount of sample packed in the rotor.

The resulting ^19^F and ^1^H NMR spectra are shown in Figure 2a,b, respectively. In both the FEC- and EC-based ^19^F NMR spectra, a signal centered around −73 ppm is observed, consistent with residual PF_6_^–^ species possibly embedded in the SEI (electrodes were washed only with DMC). Additional small resonances at approximately −80 ppm and −148 ppm for both systems are attributed to fluoro-phosphoro-oxides (“P*_x_* O*_y_* F_*z*_”)—specifically PO_2_F_2_^–^ and PO_3_F_2_^–^, which are known to arise from the partial hydrolysis of PF_6_^–^ as represented in eqs 1 and 2.

However, the main difference between the FEC- and EC-based ^19^F NMR spectra is the dominant, intense peak at ∼ −204 ppm in the FEC-based sample, assigned to LiF. This result clearly indicates the formation of a substantial inorganic LiF-rich SEI component, consistent with previous reports identifying LiF as a major FEC decomposition product.^39,55^ Similarly, Sina et al.^32^ discovered that the electrode cycled in EC/DEC/FEC is covered in a dense and uniform SEI containing mostly LiF. This decomposition is associated with the plateau at approximately 1.3 V in the first lithiation potential profile, as observed in the galvanostatic data (Figure 1a). In the FEC-based system, this LiF formation aligns with the decomposition of FEC during the first discharge, where the majority of SEI formation occurs. In contrast, the LiF signal in the EC-based material is very weak and is primarily attributed to the minor hydrolysis of PF_6_^–^ in the presence of trace water, as also suggested by the presence of PO_2_F_2_^–^ and PO_3_F_2_^–^. These species result from the reactions shown in eqs 1 and 2, which generate HF that subsequently forms LiF through a cation exchange. The ^1^H NMR spectra for both the EC- and FEC-based electrolytes confirm the presence of organic decomposition components as part of the SEI. Notably, the EC-based sample exhibits proton signals substantially more intense than those of the FEC-based sample, indicating an SEI richer in organic species for the EC-based electrolyte.

1 $$\mathrm{LiP}F_{6}+{2H}_{2}O\to \mathrm{LiP}O_{2}F_{2}+4\mathrm{HF}$$

2 $$\mathrm{LiP}O_{2}F_{2}+\mathrm{LiF}+H_{2}O\to {\mathrm{Li}}_{2}PO_{3}F+2\mathrm{HF}$$

Cross-Polarization Magic Angle Spinning NMR (CP-MAS) experiments (^1^H → ^7^Li and ^19^F → ^7^Li) were performed to resolve ^7^Li resonances from lithium sites in close spatial proximity to ^1^H and ^19^F, respectively. This approach enables the selective characterization of different components within the solid–electrolyte interphase (SEI). The selectivity arises from dipolar coupling between ^7^Li and nearby ^1^H or ^19^F nuclei, effectively serving as a spatial filter as dipolar interactions are proportional to the third power of the interatomic distances (*r* ^3^). In the CP experiment, magnetization is transferred from abundant spins (^1^H or ^19^F) to nearby ^7^Li nuclei over a defined contact time. ^7^Li signals obtained via ^1^H–^7^Li CP are therefore assigned to lithium in organic SEI species (e.g., oligomers/polymers, (CH_2_OCO_2_Li)_2_, or ROCO_2_Li), whereas ^7^Li signals polarized from ^19^F are attributed to LiF. The latter assignment is further supported by the ^19^F–^7^Li HETCOR spectra (Figure S2), in which the ^7^Li correlation signal is observed exclusively with a ^19^F resonance at around −204 ppm, consistent with the chemical shift of LiF.

### Li Ion Dynamics between the Lithiated Si/Gr and the SEI: 2D EXSY and CP-MAS EXSY NMR

Two-dimensional exchange spectroscopy (2D EXSY) MAS NMR experiments under magic-angle spinning (MAS) conditions were performed on ex-situ Si/Gr samples to directly probe potential Li^+^ exchange in equilibrium between lithiated Si/Gr and Li-ions in the SEI.

For the case of lithiated Si/Gr with a formed SEI with different Li^+^ environments, if lithium nuclei are exchanging between different environments that exhibit distinct chemical shifts in the NMR spectra within the given mixing time, such an exchange appears as off-diagonal (cross) peaks in the 2D contour plots of the 2D EXSY spectra, as shown in Figure 3a,b. As the mixing time (τ_mix_) increases, Li-ions have more time to migrate between different environments, specifically, between lithiated Si and the SEI, or between lithiated graphite and the SEI. This results in enhanced cross-peak intensity or the appearance of additional cross peaks, indicative of slower exchange processes or diffusion between more spatially separated Li-containing environments. Previous work by Gunnarsdóttir et al. employed two-dimensional exchange spectroscopy (2D EXSY) NMR to measure the lithium-ion exchange rate between metallic lithium and lithium in the electrolyte at open-circuit voltage (OCV), thereby evaluating the Li-ion permeability of the SEI in LP30 electrolyte with and without fluoroethylene carbonate (FEC).^56^ Subsequent studies have used 1D and 2D EXSY, as well as ^7^Li chemical-exchange saturation transfer (CEST) NMR, to quantify Li-ion exchange between SEI-bound lithium and metallic or plated lithium while systematically varying electrolyte composition, salt and additive chemistry, and electrochemical formation protocols.^57–59^ By comparison, interfacial lithium dynamics in silicon-based anodes has received far less attention. Here, 2D EXSY NMR was employed to investigate Li-ion exchange across the lithiated silicon (Li*_x_* Si)/SEI and lithiated graphite (Li*_x_* C_6_)/SEI interfaces. As illustrated in Figure 3a,b, cross-peaks appear exclusively between Li*_x_* Si and SEI-bound Li in both electrolyte systems (EC- and FEC-based) at a mixing time of 100 ms. This observation was further confirmed by 2D ^6^Li EXSY NMR, as shown in Figure S3. These results signify only the overall Li-ion exchange between Li*_x_* Si and the different Li-containing species in the SEI (organic and inorganic), while no exchange is detected between lithiated graphite (Li_*x*_C_6_) and the SEI for the same mixing time. Because the resonances of all SEI Li^+^ containing species overlap at ∼0 ppm, distinguishing the participation of different lithium reservoirs at the organic or inorganic species in the exchange with Li_*x*_Si is challenging.

**Figure 3:**
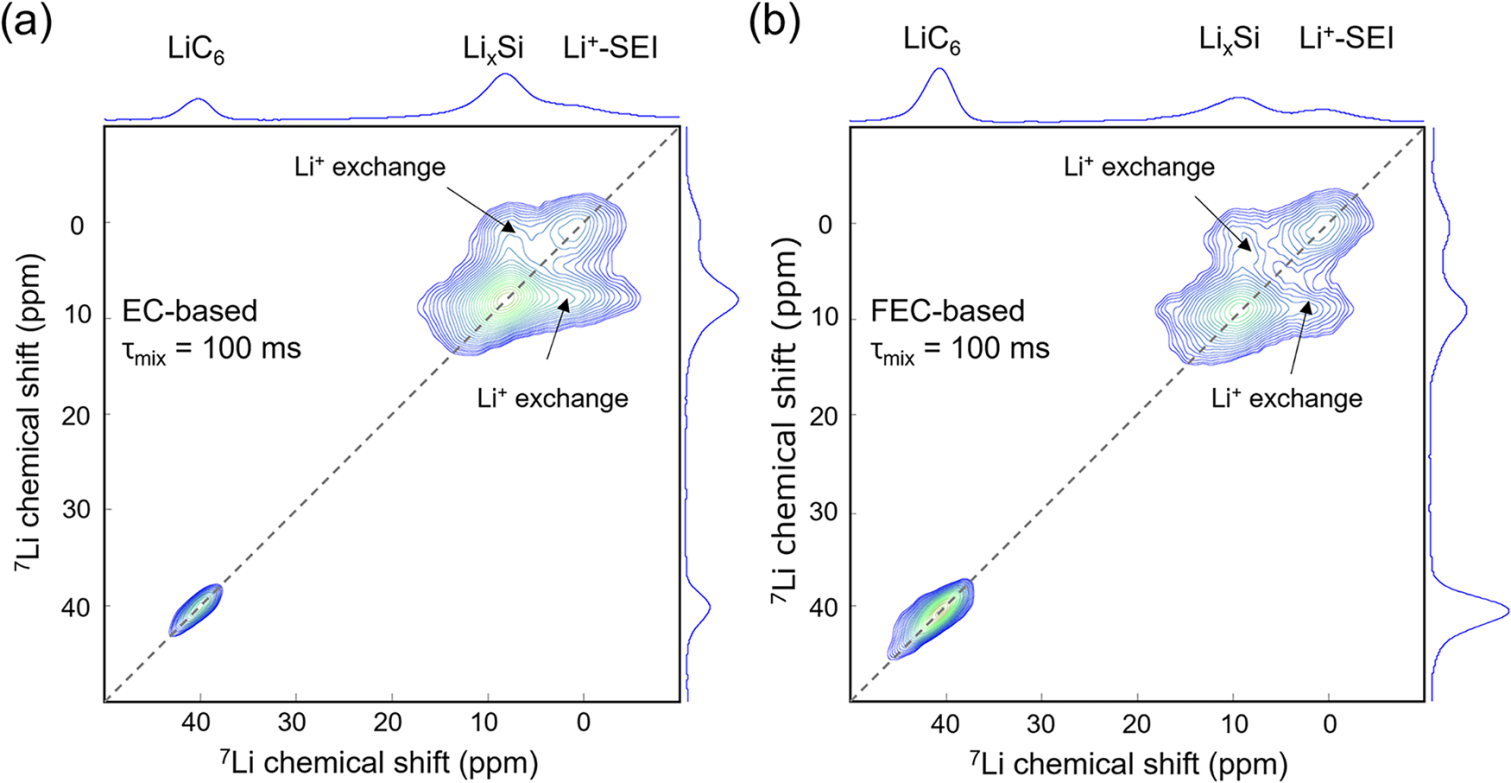
^7^Li–^7^Li EXSY NMR experiments measuring Li-ion transport between the lithiated silicon and the as-formed SEI for (a) EC-based and (b) FEC-based electrolyte.

To address this issue, we combine cross-polarization (CP) and one-dimensional EXSY, specifically ^1^H → ^7^Li and ^19^F → ^7^Li CP-MAS EXSY NMR experiments, to investigate interfacial lithium exchange at the electrode/SEI interface in lithiated anodes (Li_*x*_Si/Li_*x*_C_6_). This method enables the selective evaluation of Li-ion exchange originating either from the organic or inorganic (LiF) components of the SEI by implementing the CP step of ^1^H → ^7^Li or ^19^F → ^7^Li, respectively.

In one-dimensional (1D) CP-MAS EXSY experiments, the observation of additional resonances at increasing EXSY mixing time at the chemical shifts’ characteristic of either Li*_x_* Si, Li*_x_* C_6_, or both indicates Li^+^ transport from the SEI into the anode (Li*_x_* Si and/or Li*_x_* C_6_). This method therefore enables selective detection quantification of Li-ion transport kinetics across the SEI-anode interface from a specific SEI component. To the best of our knowledge, CP-MAS EXSY measurements targeting the SEI/anode interface have not been reported previously.

As shown in Figure 4a,b, ^1^H → ^7^Li CP-MAS EXSY experiments, and in Figure 4d, ^19^F → ^7^Li CP-MAS EXSY experiments, were carried out on both electrolyte systems to probe Li-ion dynamics associated with the organic and inorganic fractions of the SEI, respectively. At very short mixing times (∼0 ms), the ^7^Li NMR spectra recorded in both experiments are dominated by the CP signals originating from dipolar magnetization transfer from ^1^H or ^19^F, closely matching the corresponding CP-MAS spectra in Figure 2c,d. This confirms that at the start of the mixing period the observed Li nuclei reside predominantly in proximity to the organic or inorganic SEI components.

**Figure 4:**
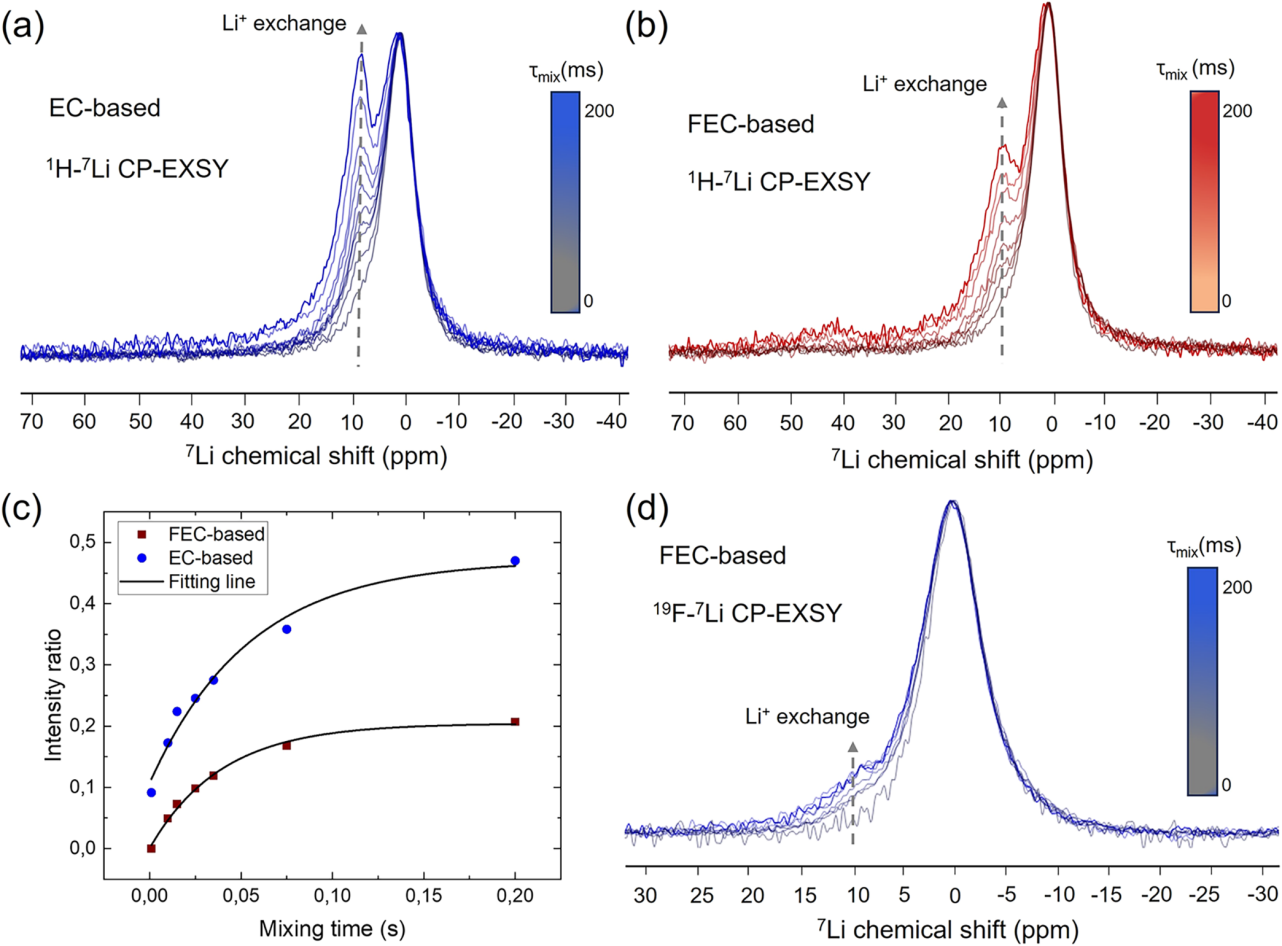
(a) and (b) ^1^H → ^7^Li CP-MAS EXSY spectra for EC-based and FEC-based lithiated Si/Gr samples, respectively. (c) Buildup curves showing the intensity ratio versus mixing time at ∼10 ppm, corresponding to Li^+^ ions migrating from the organic and inorganic SEI phases into the Si particles. Black lines represent single exponential fits used to extract exchange time constants (τ_ex_). (d) ^19^F → ^7^Li CP-MAS EXSY spectrum for the FEC-based lithiated Si/Gr sample.

As shown in Figure 4a,b, increasing the mixing time in the ^1^H → ^7^Li CP-EXSY experiments leads to the emergence of an additional resonance at approximately 9 ppm for the EC-based electrolyte and ∼11 ppm for the FEC-based electrolyte. These signals are characteristic of Li*_x_* Si species, as also observed in the 1D ^7^Li NMR spectra in Figure 1b,c. The exchange fraction at frequency corresponding to Li*_x_* Si resonance grows markedly with longer mixing times for both electrolytes, whereas they remain weak in the ^19^F → ^7^Li CP-EXSY data for the FEC system. We attribute the presence of these resonances to Li^+^ ions that were initially located within or near the organic SEI layer and subsequently diffused into the lithiated silicon phase during the mixing period. Additionally, a substantial exchange signal buildup at the Li_*x*_Si chemical shift is observed for ^1^H → ^7^Li CP-EXSY in both electrolyte-based cases (at increasing mixing times of the EXSY block of the experiment) whereas it is very minor for ^19^F -^7^Li CP-EXSY.

The nearly negligible Li-ion exchange from LiF observed in the ^19^F → ^7^Li CP-EXSY experiments agrees with the inherently low Li^+^ mobility in LiF. Previous studies report room-temperature ionic conductivities for bulk LiF in the range of ∼10^–14^ to 10^–9^ S cm^–1^.^60,61^ Conductivity measurements and molecular dynamics simulations further estimate the activation energy for Li^+^ migration to be approximately 0.55–0.73 eV^62,63^ among the highest values reported for typical SEI components. This substantial energy barrier implies that Li^+^ ions confined within the dense LiF lattice are unable to escape and reach Li*_x_* Si sites within the ≲200 ms mixing time employed here, in contrast to Li^+^ species in the more permeable, carbonate-rich organic SEI, which exhibit readily detectable exchange.

From the inspection of Figure 4b, the exchange fraction of Li from the organic Li-containing phase is much larger compared with the exchange observed for Li at the inorganic LiF phase (Figure 4d). This result demonstrates a larger fraction of Li^+^ exchange from the organic part of the SEI in the FEC-based system to the surface of the Si particles. A similar ^7^Li {^1^H → ^7^Li} CP-EXSY was conducted for the EC-based system, and the result shown in Figure 4a also clearly, showing an intense Li exchange from the organic part of SEI. No ^7^Li {^19^F → ^7^Li} CP-EXSY experiments were performed in the EC-based system, as in this case, the concentration of LiF is negligible, as shown in Figure 2a on the red spectra. The 1D version of the filtered CP-EXSY experiments allows recording the exchange process as a function of the mixing times in less time-consuming experiments as compared to the 2D EXSY versions. The ^7^Li buildup intensity corresponding to Li atoms migrating from the organic component of the SEI to the Si particles for the FEC and EC samples are shown in Figure 4c. In both cases, the data could be fitted considering single exponential functions resulting in different time constants and rates: τ_ex_ (SEI-Li_*x*_Si) = 55 ms *k*_ex_ (SEI-Li_*x*_Si) = 18 s^–1^ for EC and τ_ex_ (SEI-Li_*x*_Si) = 40 ms, *k*_ex_ (SEI-Li_*x*_Si) = 25 s^–1^ for FEC. Additionally, different fractions of exchange are observed for both electrolyte systems relative to the intensity of the main original CP signals, a larger exchange fraction is observed in the EC-based system. The observed differences in exchange fraction and rates of Li at the organic phases of FEC and EC are very valuable pieces for the description of both SEIs. In particular, a higher Li exchange fraction in EC-based indicates that the organic coating component of the SEI is larger in this case as compared to the FEC case, in agreement with EC-based system showing a relatively intense ^1^H signal in Figure 2a as compared to FEC. This conclusion is obtained from the following analysis: In the CP-EXSY experiment, the initial cross-polarization step generates ^7^Li magnetization with an intensity determined by the number of ^7^Li–^1^H dipolar pairs present in the sample—effectively proportional to the total amount of lithium-containing organic component. Assuming the chemical nature of the organic components in both EC- and FEC-based systems is similar, factors such as local dynamics and internuclear distances are expected to contribute similarly to CP efficiency. Therefore, observed differences in the exchange fractions are attributed to variations in the spatial proximity of the organic SEI to the silicon surface, specifically, to the fraction of Li^+^ in the organic phase that can access the Si surface within the mixing time window (1–200 ms). Interestingly, although the calculated exchange rate is faster in the FEC-based SEI, the exchange fraction is smaller compared to the EC-based system. This suggests that a larger portion of the organic phase in the FEC-based SEI is located farther from the Si surface, limiting the extent of Li^+^ exchange. This interpretation aligns with the presence of a substantial LiF-rich inorganic component in the inner layer of the formed SEI with FEC, which likely acts as a physical barrier and prevents the organic phase from uniformly coating the silicon particles—unlike in the EC-based system. This scenario is supported by the previous operando soft X-ray absorption spectroscopy (sXAS) study, which has shown that in FEC-containing electrolytes, LiF forms directly on the a-Si electrode surface at higher potentials, while organic SEI components subsequently nucleate on top of the LiF layer.^28^ Our ^19^F NMR data (Figure 2a) further confirm this interpretation, revealing a substantial LiF fraction in the FEC-based sample, whereas LiF is virtually absent in the EC-based system. This difference accounts for the larger ^1^H → ^7^Li CP-EXSY exchange fraction observed with the EC electrolyte: the predominately organic SEI in the EC-based system remains in direct contact with the silicon surface, facilitating more interfaces for Li exchange, while in the FEC-based system the LiF layer spatially separates the organic overlayer from the a-Si, suppressing exchange.

The relatively faster exchange of Li-ions in the SEI/anode interface of FEC agrees with a better performance of this electrolyte and reveals a faster Li diffusivity of lithium through the SEI, which is a key parameter in the SEI performance. Even though 2% VC additive is present in the FEC-based electrolyte system used in this work, its contribution to the SEI is expected to be minor compared to the 45% FEC cosolvent. Based on previous study, the organic part of the SEI formed in FEC- and VC-containing electrolytes is reported to contain a significant amount of branched/cross-linked PEO-type polymeric species, whereas additive-free carbonate electrolytes (EC-based) typically yield more linear PEO-type components.^55^

### Correlating MAS NMR and Electrochemical Data for Extended Cycling: Insights into Failure Mechanisms

The voltage profiles and differential capacity (d*Q*/d*V*) plots of the Si/Gr composite anode cycled in EC-based and FEC-based electrolytes in different cycles are presented in Figure 5a–d. In the first 10 cycles, the Si/Gr anodes maintained their lithiation/delithiation capacities for both EC- and FEC-based electrolytes. However, upon further cycling, the reversible capacity of the cell with the EC-based electrolyte system began to decrease continuously relative to its initial capacity value. In contrast, the cell with the FEC-based electrolyte kept stable capacity values over all the 49 cycles. Additional insights into the mechanisms behind these capacity changes can be obtained by examining the evolution of the voltage profiles and dQ/dV curves for both systems, as shown in Figure 5b,d, since shifts in redox potential and their corresponding capacity contributions can indicate the thermodynamic or kinetic events occurring during the electrochemical reactions. The differential capacity (d*Q*/d*V*) profiles exhibit two broad lithiation peaks attributable to silicon at approximately 230 and 100 mV vs Li/Li^+^. The lower-potential feature partially overlaps the two sharp graphite lithiation peaks. As detailed in previous reports,^28^ these signals arise from successive amorphous Li_*x*_Si phase transformations. Specifically, upon lithiation, the ∼230 mV peak marks the formation of a-Li_*x*_Si with *x* ≤ 2 (up to a-Li_∼2_Si), whereas the ∼0.10 V peak corresponds to further alloying to 2 ≤ *x* ≤ 3.75 (up to a-Li_3.5–3.75_Si). During delithiation, two broad peaks appear at ∼270 and ∼470 mV. These reflect the reverse transformations of amorphous lithium silicide phases, namely, a-Li_3.5–3.75_ Si → a-Li_2_Si and a-Li_2_Si → a-Si, respectively.

**Figure 5:**
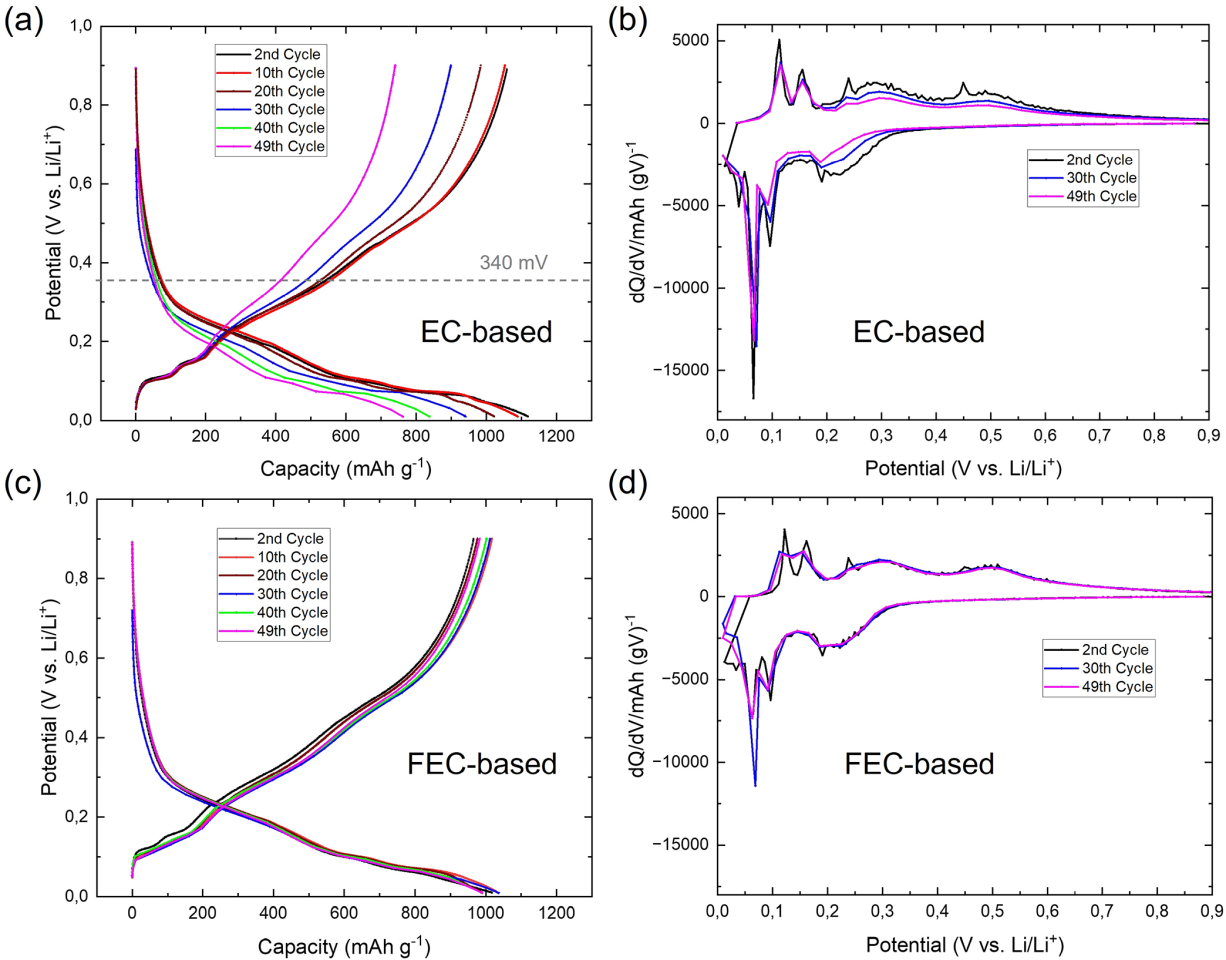
Voltage profile of Si/Gr anodes in cells with (a) EC-based and (c) FEC-based electrolyte. C rate = C/15. Cycle: 2, 10, 20, 30, 40, and 49. (b, d) Corresponding differential capacity plots (d*Q*/d*V*) of cycles 2, 30 and 49.

It can be observed that the silicon lithiation peaks in electrodes cycled with EC-based electrolytes gradually weaken over the course of 49 cycles, as shown in Figure 5b for the second, 30th, and 49th cycles. In contrast, for cells cycled with FEC-based electrolytes, the silicon lithiation/delithiation peaks are maintained throughout all 49 cycles, as depicted in Figure 5d for the same cycles. This behavior is consistent with the improved cycling performance observed for the FEC-based systems. Interestingly, even though the Si/Gr composite anode with the EC-based system exhibits capacity fade upon continued cycling, the graphite-derived capacity remains essentially constant throughout all 49 cycles, as evidenced by the maintained plateaus at approximately 110, 150, and 230 mV in the voltage profile. This observation indicates that in the EC-based system the capacity fading in the Si/Gr composite originates exclusively from the silicon component. Moreover, no significant shift in the voltage profile was observed during lithiation and delithiation—unlike other negative electrodes such as graphite—where such shifts accompany capacity fading. Typically, these shifts in potential profiles are attributed to increased cell polarization, which causes the measured cell potential to reach the cutoff voltage before the electrode reaches its true thermodynamic potential.

However, Figure 5a shows that for the Si/Gr anode in the EC-based electrolyte, the most pronounced changes in the delithiation profiles appear at comparatively high potentials. This behavior is consistent with earlier studies, which attribute the loss of reversible capacity primarily to the Li poor silicide phase at these higher delithiation potentials, namely, to incomplete delithiation and the associated Li^+^ trapping in Li_*x*_Si. Specifically, the potential profile below ≈340 mV—assigned to the delithiation of the Li-rich silicon phase (Li_3.5–3.75_Si) together with Li_*x*_C_6_, undergoes only minor changes. The capacity associated with these low-potential plateaus during both lithiation and delithiation remains essentially constant, particularly for the first 20 cycles. By contrast, the potential profile above ≈340 mV, which corresponds to the Li-poor amorphous silicon phase (≈Li_2_Si), shows a pronounced decline in its capacity contribution as cycling progresses. This observation suggests that most of the capacity fade in EC-based Si/Gr anodes arises from incomplete delithiation of this Li-poor phase, consistent with previous reports on silicon anodes.^64^ Starting from the 20th cycle, the entire voltage profile corresponding to delithiation of lithiated amorphous silicon begins to change, including the regions below ∼340 mV associated with delithiation of the Li-rich phase (∼Li_3.5–3.75_Si). However, the delithiation plateaus attributed to graphite (Li_*x*_C_6_) persist. This indicates that beyond the 20th cycle, the lithiation process involves partially lithiated silicon (∼Li_2_Si), possibly inactive rather than pure amorphous silicon, since the electrode is not fully delithiated during the previous cycle. One possible reason for the capacity fading during delithiation at relatively higher potential when cycling with EC-based electrolyte is that the volume change, specifically contraction, of Si particles during cycling can lead to electronic and ionic disconnection of the silicon particles, creating inactive lithiated Si particles. This is consistent with previous work suggesting that the greatest mechanical stress is expected to occur toward the end of delithiation, due to the contraction of a-Li_*x*_Si.^28^ This mechanical stress is overall detrimental for the formed SEI on the Si particle, especially for EC-based system with organic rich SEI which is has a low interfacial energy with Si surface leading to strong adhesion that could easily break with the mechanical stress unlike the inorganic rich (LiF) for FEC-based, where the LiF is a main part of the inner SEI which has low adhesion as well as a better mechanical strength which keeps the particle intact and contained.^35,65^

For further analysis, Figure 6a presents the capacity and Coulombic efficiency versus cycle number for cells using EC- and FEC-based electrolytes. After approximately 20 cycles, both the Coulombic efficiency and discharge capacity of the cell containing the EC-based electrolyte begin to decline, with the discharge capacity falling below 750 mAh g^–1^ by cycle 50—compared to a delithiation capacity of 1119 mAh g^–1^ in the second cycle. In contrast, the cell with the FEC-based electrolyte maintains both high Coulombic efficiency and stable capacity throughout the 50 cycles, retaining a discharge capacity of 985 mAh g^–1^, close to its second-cycle capacity of 1017 mAh g^–1^. These results clearly demonstrate the stabilizing effect of FEC as an additive/cosolvent in enhancing the long-term cycling performance of Si/graphite composite anodes.

**Figure 6:**
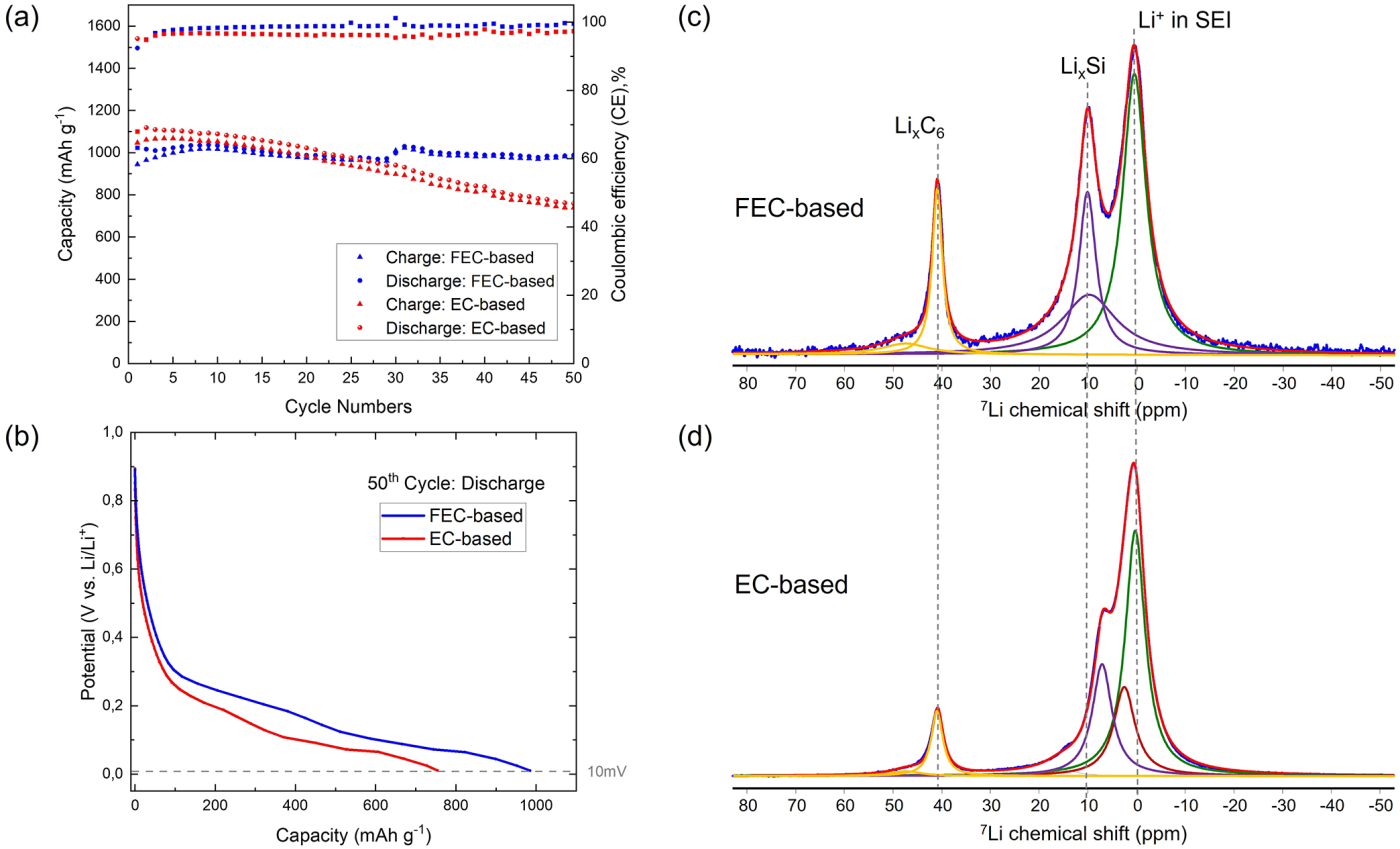
(a) Specific capacity versus cycle number for Si/Gr anodes cycled with EC-based (red) and FEC-based (blue) electrolytes. (b) Voltage profiles of the Si/Gr anode during the 50th lithiation cycle in EC- and FEC-based electrolytes, with a lower cutoff potential of 10 mV—the state at which the NMR experiments were performed. (c, d) ^7^Li MAS NMR spectra of the Si/Gr anode discharged to 10 mV using EC-based (c) and FEC-based (d) electrolytes.

To complement the observed capacity decay in the EC-based system and the capacity retention in the FEC-based system, electrodes extracted after 50 cycles were characterized by ex-situ ^7^Li NMR. The ^7^Li NMR spectrum of the FEC-based system (Figure 6c) closely resembled that of the first discharge (Figure 1b), indicating minimal changes in lithium distribution and supporting the observed electrochemical stability. In contrast, the EC-based system exhibited significant differences between the ^7^Li NMR spectrum after the first cycle (Figure 1c) and that after the 50th cycle (Figure 6d), reflecting substantial changes in the bulk lithium environment consistent with capacity fading and electrode degradation. A detailed analysis of the ^7^Li NMR spectrum after the 50th cycle required the inclusion of an additional component, indicating the emergence of a distinct lithium environment—one more than the three-component model used to describe the first discharge. This additional resonance, appearing at around ∼3 ppm, is likely associated with a new Li-containing phase, attributed to trapped lithium within the bulk silicon. Li^+^ trapping has been widely proposed as a major contributor to capacity loss in Si-based anodes, predominantly occurring during delithiation.^13,29,33,66–69^ In contrast, the ^7^Li NMR spectrum of the FEC-based system after 50 cycles could still be accurately fitted using the same three-component model as in the first cycle, indicating that the lithium distribution remained largely unchanged throughout the cycling.

Further inspection of the silicide region in the EC-based sample revealed two notable lithium environments: (i) Li near isolated silicon atoms, centered around ∼6 ppm, consistent with a stoichiometry close to Li_3.5–3.75_Si, and (ii) Li in proximity to larger Si clusters or extended silicon networks, resonating at 2–4 ppm, corresponding to a stoichiometry similar to Li_2_Si. The latter environment—present only in the EC-based system—would not be expected at the discharge potential cutoff of 10 mV used for ex-situ NMR, suggesting that these Li_2_Si-like phases originate from particles that are either electrically isolated from the current collector or exhibit sluggish delithiation kinetics. To validate this interpretation, the relative contributions of distinct lithium environments were quantified from the ^7^Li NMR spectra and compared to the electrochemical data for both electrolyte systems. The lithium environments corresponding to Li in graphite (Li_*x*_C_6_, ∼41 ppm) and Li in isolated silicon environments (Li_3.5–3.75_Si, ∼6 ppm) were associated with a reversible capacity at the given lithiation state. In contrast, Li^+^ environments appearing at ∼ 3 ppm, consistent with previous assignments by Key et al.,^42^ were attributed to larger Si clusters associated with irreversible capacity due to Li^+^ trapping. By calculating the intensity ratio of Li^+^ in the reversible environments (Li_*x*_C_6_ + Li_3.5–3.75_Si) to the total bulk lithium (Li_*x*_C_6_ + Li_3.5–3.75_Si + Li_2_Si), the reversible lithium content at the 50th cycle can be estimated from NMR. This NMR-derived ratio (∼0.64) agrees well with the electrochemically determined capacity retention (∼0.67, i.e., 50th cycle capacity relative to the second), demonstrating excellent consistency between structural and electrochemical measurements.

Applying the same analysis to the FEC-based system after 50 cycles, the ^7^Li NMR spectra were deconvoluted into three main components at ∼41 ppm (Li_*x*_C_6_), ∼10 ppm (Li near isolated Si), and ∼0 ppm (Li in SEI). No signals corresponding to trapping Li^+^ (Li_2_Si) in larger Si clusters were observed, likely due to their absence or concentrations below the detection threshold. The presence of only reversible Li^+^ environments (Li_*x*_C_6_ + Li_3.5–3.75_Si) aligns with the nearly 100% capacity retention observed electrochemically between the second and 50th cycles. This strong correlation further supports the conclusion that the FEC-based system effectively suppresses Li^+^ trapping, enabling stable cycling with minimal degradation. To the best of our knowledge, this work provides the first direct solid-state NMR evidence of Li^+^ trapping in Si-based composite anodes. Although previous operando ^7^Li ssNMR studies have reported Li^+^ trapping in silicon anodes and proposed the accumulation of trapped lithium–silicide phases as a mechanism for capacity fading, these studies were performed in full cells under different conditions (and with Si-only anodes). Nevertheless, our ex-situ NMR observations for Si/graphite composite anodes in EC-based electrolyte are qualitatively consistent with this mechanism, showing accumulation of a trapped lithium–silicide component upon extended cycling.^70,71^

### Quantitative Analysis of Irreversible Capacity: Contributions from SEI Formation and LixSi Trapping

While previous studies have linked capacity fade during extended cycling of Si-based anodes to continuous SEI growth/electrolyte decomposition during lithiation and to lithium trapped in Li*_x_* Si after delithiation, quantitative analysis has largely been limited to reporting the total capacity loss (*Q*(tot)_irr_) as a function of cycle number (*n*), based on galvanostatic cycling data.^26,69^ To the best of our knowledge, no quantitative method has been available to extract the separate contributions of continuous SEI growth and Li^+^ trapping as a function of cycle number directly from the galvanostatic data. Here we introduce a set of cycle-resolved expressions (eqs 3–6) that partition the irreversible capacity of every cycle into two distinct components: one arising from SEI formation and the other from residual/trapped Li_*x*_Si. The derivation rests on two experimentally supported assumptions: (i) SEI formation occurs predominantly during the lithiation, and (ii) lithium becomes trapped in the silicon host only during the subsequent delithiation step, when a fraction of Li remains in the amorphous phase. By assigning SEI growth to a part lithiation region and Li^+^ trapping to a part delithiation region, the framework decouples the two mechanisms and quantitatively accounts for every irreversibly lost charge as presented below from eq 3–6.

3a $${Q\;\left(tot\right)}_{irr}=\sum_{i=1}^{50}\left(Q_{i}^{lithiation}-Q_{i}^{delithiation}\right)$$

3b $$\sum_{i=1}^{50}Q\;\left(SEI\right)={(Q}_{1}^{lithiation}-Q_{1}^{delithiation})+\sum_{i=2}^{50}\left(Q_{i}^{lithiation}-Q_{i-1}^{delithiation}\right)$$

3c $$\sum_{i=2}^{50}Q\;(trapped\;LixSi)=\sum_{i=2}^{50}{(Q}_{i-1}^{delithiation}-Q_{i}^{delithiation})$$

3d $${Q\;\left(tot\right)}_{irr}=\sum_{i=1}^{50}Q\;\left(SEI\right)+\sum_{i=2}^{50}Q\;(trapped\;LixSi)$$

eqs 3a–d provides an explicit way to separate the total irreversible capacity, Q­(tot)_irr_, into two underlying mechanisms: (a) lithium consumed by the SEI and (b) lithium trapped in the silicon phase (Li_*x*_Si) that does not fully extract during delithiation. This first eq 3 sums up the difference between the lithiation and delithiation capacities over multiple cycles (here, 50). Any capacity lost in each cycle, i.e., capacity that cannot be recovered on delithiation—contributes to the overall irreversible capacity.

eq 4 isolates the capacity consumed by SEI formation and growth. The first cycle often involves substantial SEI formation (*Q*_1_^lithiation^ – *Q*_1_^delithiation^), while subsequent cycles may exhibit smaller but cumulative SEI growth (the summation term). Equation 5 represents the capacity associated with lithium that remains in the silicon host from one cycle to the next (incomplete delithiation). Over repeated cycles, this residual Li_*x*_Si phase contributes to a progressively growing fraction of the total irreversible capacity. Finally, eq 6 states that the total irreversible capacity can be viewed as the sum of the SEI-related contribution plus the capacity lost to trapped lithium in the silicon. This unified expression underscores how both surface reactions (SEI formation and growth) and incomplete delithiation (trapped Li_*x*_Si) must be considered to accurately quantify the total irreversible losses over multiple cycles. A full derivation is provided in the Supporting Information.

As shown in Figure 7, the cumulative irreversible capacities Q­(tot)_irr_, ∑Q­(SEI), and ∑Q­(trapped Li_*x*_Si) are plotted as a function of cycle number over 50 cycles, calculated using eqs 3, 4, and 5, respectively, for cells employing EC- and FEC-based electrolytes. For both electrolytes, *Q*(tot)_irr_ and ∑*Q*(SEI) begin at approximately 495 mAh g^–1^ (EC) and 550 mAh g^–1^ (FEC), reflecting substantial SEI formation during the first cycle. These quantities continue to increase with further cycling, indicating that irreversible processes persist beyond the initial formation stage. During the first 8–10 cycles, the slopes of *Q*(tot)_irr_ and ∑Q­(SEI) are nearly identical for both electrolyte systems, suggesting that the total irreversible capacity in this early regime is dominated almost entirely by ongoing SEI growth. However, starting around the 10th cycle, the slopes of *Q*(tot)_irr_ and ∑*Q*(SEI) for the EC-based system become substantially steeper, reflecting a more pronounced increase in the irreversible capacity relative to the FEC-based system. During the first 10 cycles, both EC- and FEC-based cells show no noticeable rise in the ∑*Q*(trapped Li_*x*_Si), indicating negligible lithium trapping within the silicon particles at this stage. Beyond the 10th cycle, ∑Q­(trapped Li_*x*_Si) in the EC-based cells begins to increase, while the FEC-based cells remain essentially unchanged, suggesting that Li^+^ trapping is effectively suppressed in the presence of FEC. Notably, the onset of Li^+^ trapping in the EC-based cells coincides with the beginning of capacity fading, as observed in Figure 5a, whereas the FEC-based cells maintain stable capacity throughout the cycling period.

**Figure 7:**
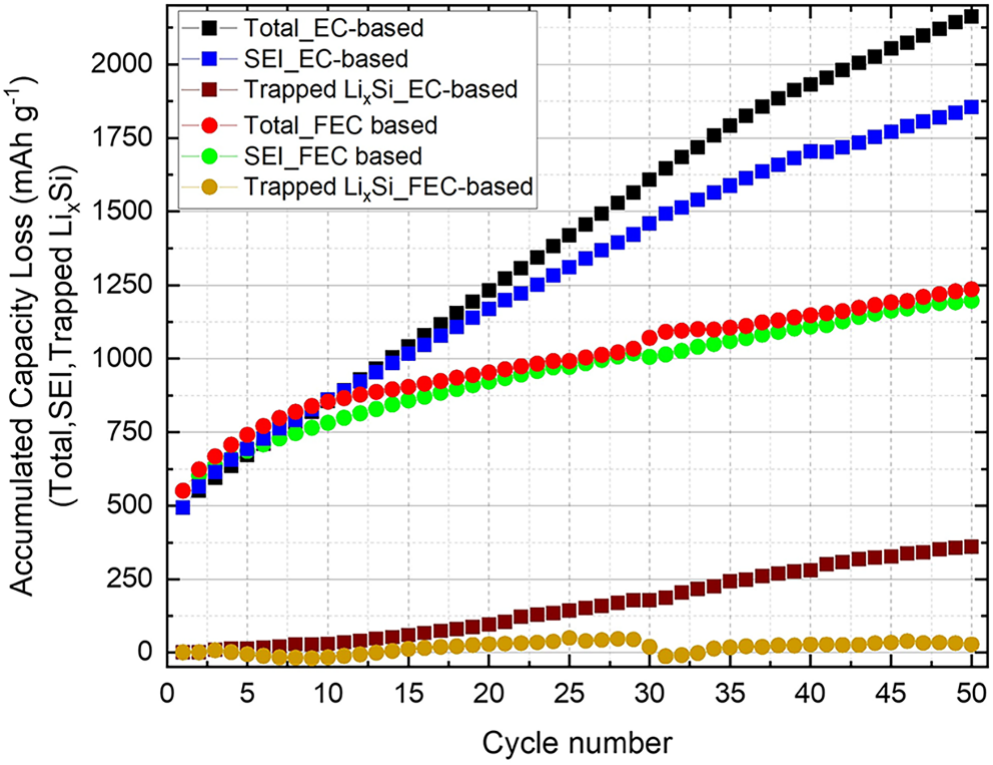
Accumulated capacity loss for total, SEI, and trapped Li_*x*_Si, as defined by eqs 3, 4, and 5, respectively, is shown as a function of cycle number. These data are obtained from galvanostatic cycling for both EC-based and FEC-based electrolytes.

The cumulative SEI-related irreversible capacities reached 1855 mAh g^–1^ for the EC-based system and 1236 mAh g^–1^ for the FEC-based system over 50 cycles. In agreement with previous studies, the continuous and thicker SEI formed in the EC-based electrolyte likely imposes a higher charge-transfer resistance, thereby hindering the complete delithiation of silicon and promoting the formation of inactive (trapped) Li_*x*_Si. In contrast, the stable SEI formed in the FEC-based system preserves the electrochemical accessibility of the Si particles throughout cycling.^64,68,69^ This is supported by the additional irreversible capacity loss of up to 360 mAh g^–1^ in the EC-based cells, which corresponds to a true loss of active capacity from the anode due to Li_*x*_Si trapping. In contrast, the FEC-based system exhibits virtually no evidence of Li^+^ trapping. A previous investigation by Bao et al.^33^ on Si thin-film anodes employed titration-gas chromatography (TGC) to quantify and isolate the contributions from continuous SEI formation (*Q*_SEI_) and trapped Li–Si phase (*Q*_trapped Li_) during extended cycling. Interestingly, their plots showing the evolution of the relative capacities of SEI and trapped Li–Si alloy versus cycle number exhibit a trend remarkably similar to that derived from our galvanostatic analysis. Similarly, Zhang et al.^68^ conducted a TGC study on nanoparticle-based Si anodes using the conventional LP30 electrolyte, with and without FEC. They quantified the evolution of SEI-associated Li^+^ and the inactive (trapped) Li_*x*_Si during extended cycling. Their results demonstrated that the electrolyte containing FEC significantly reduced the accumulation of inactive Li_*x*_Si compared to that of the FEC-free system. These consistent observations from both works further support the validity of our analytical approach in decoupling SEI growth and Li^+^ trapping contributions from the galvanostatic cycling data.

### Lithium Redistribution in the Si/Gr Composite Anode: Lithiation Transfer from Gr to Si

Several studies have reported that incorporating graphite (Gr) into Si-based anodes offers advantages in terms of maintaining the structural integrity of the electrode. However, other reports have highlighted a complex interplay between lithiated Si and Gr, which can result in a lithium redistribution between the two components in the composite anode. This redistribution may lead to overlithiation of the Si phase, particularly during lithiation. Such overlithiation is detrimental to Si-based systems, as it exacerbates mechanical stress on the Si particles, accelerating electrode degradation. Importantly, this phenomenon is driven by equilibrium potential differences between the two phases and can occur, irrespective of the electrolyte formulation.

To investigate whether such lithium redistribution continues to evolve postcycling, all the samples used in this study—both EC- and FEC-based extract for NMR measurement after the first and 50th discharge—were remeasured repeatedly in a range of 10 days to monitor whether the lithium distribution changed over time and to assess any potential degradation using the ^7^Li MAS NMR spectra. Interestingly, a consistent trend was observed across all samples, regardless of electrolyte type or the number of cycles, as shown in Figure 8a–d.

**Figure 8:**
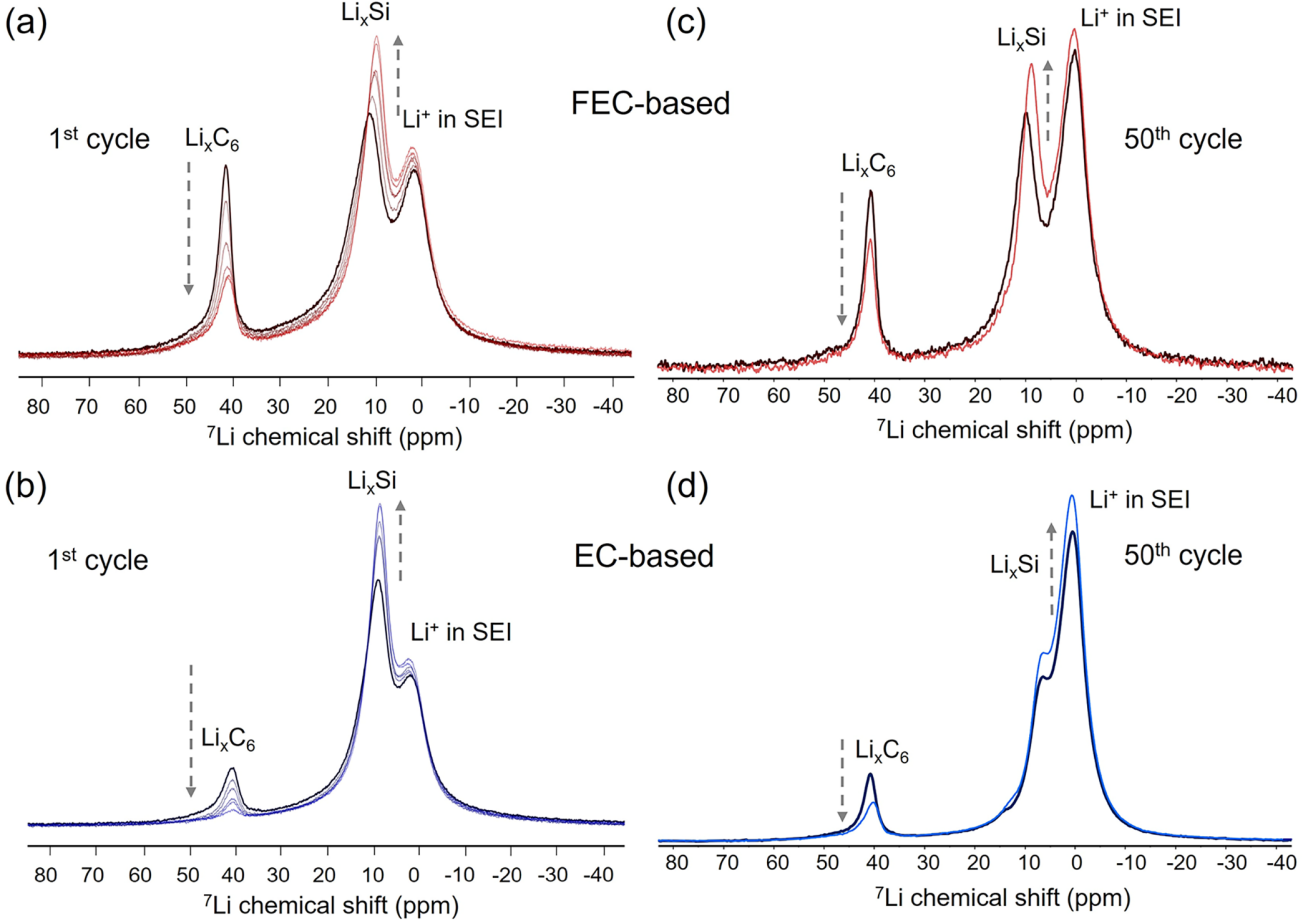
Evolution of ^7^Li NMR spectra in the range of 10 days for lithiated Si/Gr samples after the first cycle with FEC-based (a) and EC-based (b) electrolytes and after 50 cycles with FEC-based (c) and EC-based (d) electrolytes. Note: The spectra shown were acquired and reacquired on different days to illustrate the time-dependent evolution of the ^7^Li NMR spectra; they are not intended for direct quantitative comparison between electrolyte systems, but rather to show that this behavior occurs regardless of the electrolyte when the composite electrode is in the lithiated state.

A systematic decrease in the peak intensity around 42 ppm, corresponding to lithium in stage-1 graphite (LiC_6_), was observed across all the remeasurements. This persistent decline suggests the continuous delithiation of lithiated graphite during storage within the NMR rotor.

In contrast, the intensity of the signals corresponding to lithiated silicon (Li*_x_* Si) increased over time. This increase was accompanied by a gradual shift to lower chemical shifts, indicating further lithiation of the silicon phase. Taken together, these changes provide strong evidence of directional lithium redistribution from graphite to silicon occurring spontaneously during storage. Recently, Frankenstein et al.^72^ reported a similar observation by mixing lithiated graphite with crystalline Si (c-Si) powder. Using ^7^Li NMR to follow the evolution over time, they observed a clear decrease in the intensity of the ^7^Li signal assigned to LiC_6_ (≈43 ppm), accompanied by the emergence of a new ^7^Li resonance in the 15–12 ppm range, corresponding to lithiated silicon (Li*_x_* Si). Another study by Heubner et al.^73^ demonstrated internal Li redistribution in Si/graphite blend electrodes using a special electrochemical setup. After lithiation, upon interruption of the external load, they observed a negative current at the Si electrode, indicating a reduction reaction, while simultaneously an oxidative current at the graphite electrode was observed that exactly compensated for the Si current. This behavior indicates that Li is extracted from graphite and inserted into Si during the relaxation period.

This spontaneous overlithiation may have detrimental consequences. Excessive lithiation can lead to the formation of crystalline Li_15_Si_4_ and additional volume expansion, both of which are associated with mechanical degradation and capacity loss. Although in silicon-based anodes it is common practice to avoid deep lithiation by limiting the lower cutoff potential, this strategy may be insufficient in Si/graphite composite systems. As graphite delithiates over time, it effectively acts as a lithium reservoir, enabling unintended lithiation of silicon even under open-circuit potential (OCP) conditions. This finding highlights a potential failure mechanism in Si/Gr composite electrodes and emphasizes the need for further strategies to prevent lithium redistribution and overlithiation during rest time in the lithiation state.

## Conclusion

We compared Si/graphite composite anodes cycled in two different electrolytes—one containing FEC and the other based on conventional EC—to investigate SEI composition, interfacial dynamics, and their correlation with electrochemical performance. This study combined galvanostatic cycling with ex-situ multinuclear MAS NMR (^7^Li, ^19^F, ^1^H), performed on cycled anodes after the first cycle and after 50 cycles, in the lithiated state. The ^7^Li MAS NMR spectra obtained after the first lithiation revealed a similar lithium distribution across lithiated graphite (Li*_x_* C_6_), lithiated silicon (Li*_x_* Si), and lithium in the SEI, comparing the two electrolyte systems. Moreover, the results from 1D ^1^H and ^19^F NMR, together with ^1^H/^19^F–^7^Li cross-polarization (CP) and ^1^H/^19^F–^7^Li HETCOR spectra, showed that the FEC-based electrolyte forms an inorganic-rich LiF-dominated SEI, whereas the EC-based electrolyte yields a more organic-rich SEI.

Following the identification of SEI compositions formed in both electrolytes, we performed 2D ^7^Li EXSY experiments to investigate Li-ion exchange across the SEI/lithiated-anode interface. The 2D ^7^Li EXSY spectra revealed Li^+^ exchange between Li*_x_* Si and the SEI in both electrolyte systems. To further elucidate the origin of this exchange, we employed ^1^H/^19^F–^7^Li CP-EXSY experiments, which demonstrated that the Li^+^ exchange occurs almost entirely through the organic fraction of the SEI, with only a minor contribution from the inorganic LiF layer in the FEC-based electrolyte. Analysis of the CP-EXSY spectra across various mixing times enabled the extraction of the exchange rate constant (*k*_ex_) for both systems. The FEC-based electrolyte exhibited a relatively faster exchange rate, whereas the EC-based system showed a larger fraction of Li^+^ participating in the exchange, originating predominantly from the organic SEI phase—consistent with a thicker organic interphase in direct contact or close proximity to the lithiated Si particles.

After 50 cycles, the ^7^Li MAS NMR spectrum of the FEC-based system showed a lithium distribution comparable to that of the first cycle, indicating minimal structural or chemical changes. In contrast, the EC-based system exhibited an overall decrease in the Li*_x_* Si signal intensity, a shift toward lower parts per million (ppm), and the presence of an additional component attributed to Li_2_Si. These changes indicate incomplete delithiation (Li^+^ trapping), with fewer Si particles remaining electrochemically active and the Si that did participate in the electrochemistry being in a more lithiated state compared to the first cycle. To further interpret these observations, we conducted a detailed analysis of the potential profiles across multiple cycles and introduced a set of cycle-resolved equations (eqs 3–d). This quantitative framework enables partitioning of the irreversible capacity in each cycle into two distinct contributions: continuous SEI growth and Li^+^ trapping within Li*_x_* Si.

Overall, the results demonstrate that an inorganic-rich, LiF-dominated SEI (promoted by FEC) limits parasitic side reactions, maintains fast Li-ion exchange at the SEI/lithiated anode interface, suppresses Li^+^ trapping, and thereby preserves the capacity for extended cycling. In contrast, the organically rich SEI formed in the EC-based electrolyte drives continuous electrolyte reduction, extensive Li^+^ trapping, and rapid capacity decay. These findings underscore the importance of carefully selecting an appropriate electrolyte formulation when working with Si-based composite anodes.

## Supplementary Material



## Notes

Additional experimental details; equations; the CP-MAS EXSY pulse program; and supporting NMR figures, including galvanostatic lithiation (discharge) profiles, ^19^F–^7^Li HETCOR spectra, ^6^Li–^6^Li EXSY spectra, and ^1^H, ^19^F, and ^7^Li NMR spectra (PDF)
