# Supplementary material for: Solid-State NMR Investigation of Electrolyte Effects on Silicon–Graphite Composite Anode: Solid Electrolyte Interphase Formation and Failure Mechanisms
Source: Chem Mater. 2026 Apr 23;38(9):4450–65. doi: 10.1021/acs.chemmater.5c02476 (PMC13173503; doi:10.1021/acs.chemmater.5c02476)
Supplement: Supplementary file 1 [file cm5c02476_si_001.pdf]

Supporting information

Solid-State NMR Investigation of Electrolyte Effects on Silicon–Graphite Composite Anode: Solid Electrolyte Interphase Formation and Failure Mechanisms

Nahom Enkubahri Asres[a,b,c], Marta Cabello[a], Muhammad Khurram Tufail[a], Kerman Gomez Castresana[a], Aitor Villaverde[a], Juan Miguel López del Amo[a*]

[a]Centre for Cooperative Research on Alternative Energies (CIC energiGUNE), Basque Research and Technology Alliance (BRTA), Alava Technology Park, Albert Einstein 48, Vitoria-Gasteiz 01510, Spain

[b]Departament of Organic and Inorganic chemistry, Faculty of Science and Technology, University of the Basque Country, UPV/EHU, Bº Sarriena s/n, 48940 Leioa, Spain

[c]Division 6.3 Structure Analysis, Bundesanstalt für Materialforschungund -prüfung (BAM), Unter den Eichen 87 12203, Berlin, Germany

**E-Mail: jmlopez@cicenergigune.com*

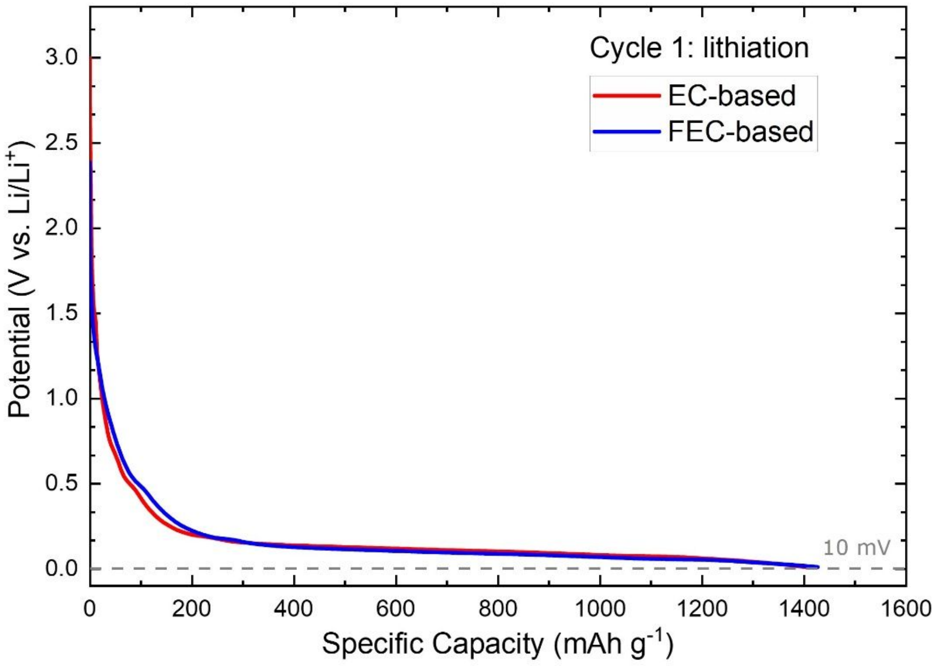

Figure S1. Galvanostatic lithiation (discharge) profile for EC- (red) (a) and FEC-based (blue) electrolyte with a lower potential cut-off of 10 mV.

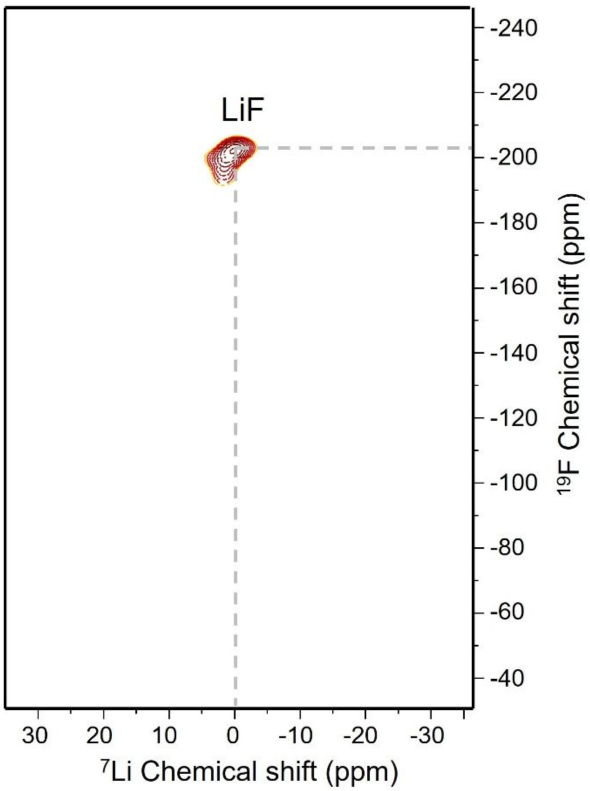

Figure S2. ^{19}F–^{7}Li HETCOR spectra of the FEC-based electrolyte system for a lithiated Si–graphite composite anode extracted after the first cycle (discharged to 10 mV).

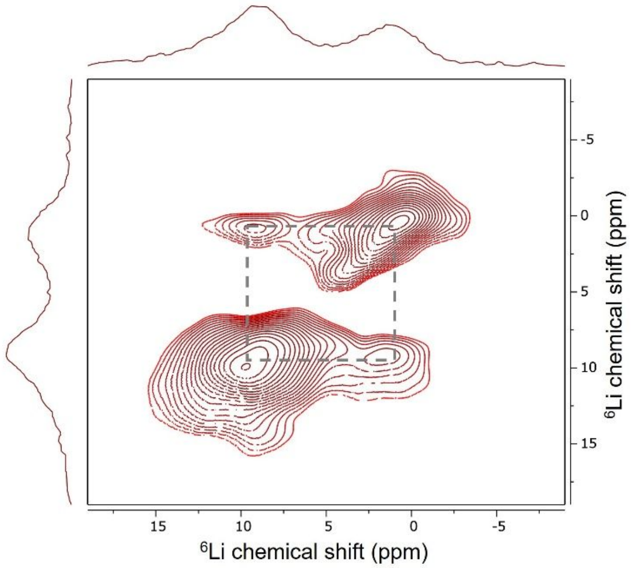

Figure S3. ^{6}Li-^{6}Li EXSY spectra of the FEC-based electrolyte system for a lithiated Si–graphite composite anode extracted after the first cycle (discharged to 10 mV).

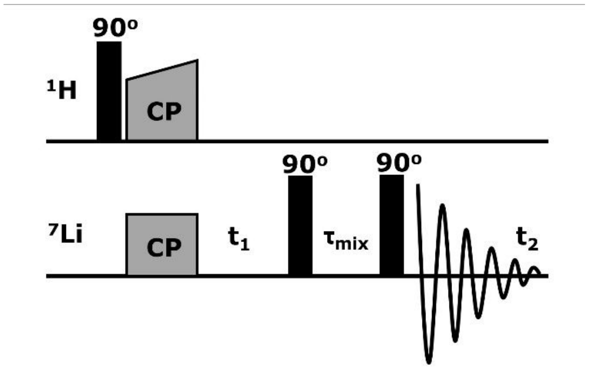

Figure S4. CP-MAS EXSY pulse program

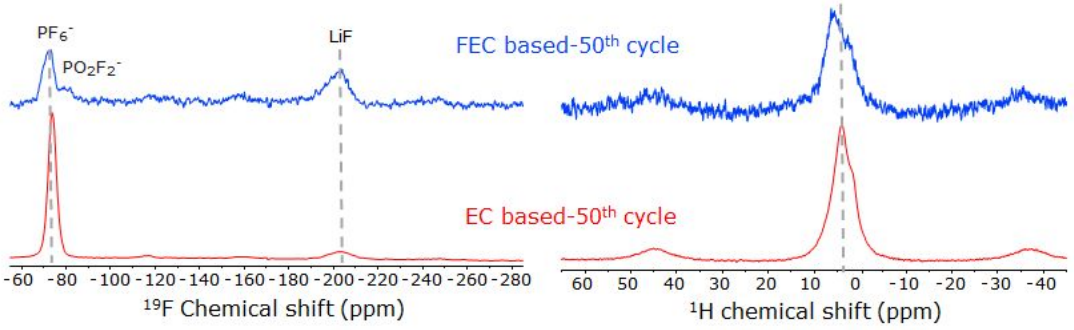

Figure S5. ^{1}H and ^{19}F NMR spectra after 50 cycles for FEC- and EC-based electrolyte

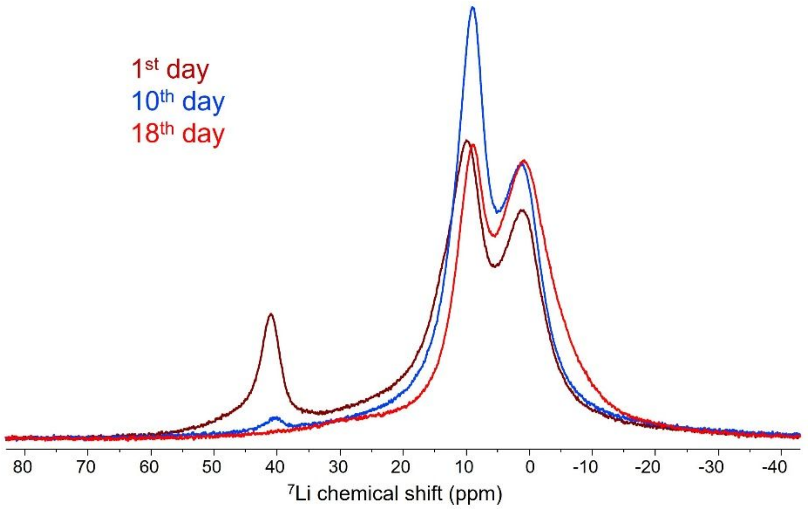

Figure S6. Evolution of ^{7}Li NMR spectra for another lithiated Si/Gr sample for FEC-based electrolyte after 1st lithiation

Proof that ΣQ(SEI) + ΣQ(LixSi) = $Q(tot)_{irr}$

This derivation shows that the SEI term (Eq 3b) and the Li_xSi-trapping term (Eq 3c) add up exactly to the total irreversible capacity (Eq 3a) .

1. Definitions

Eq 3a – Total irreversible capacity:

$$Q\ (tot)_{irr} = \sum_{i=1}^{50}\left(Q_i^{lithiation} - Q_i^{delithiation}\right)$$

Eq 3b – SEI-related capacity:

$$\sum_{i=1}^{50} Q(SEI) = \left(Q_1^{lithiation} - Q_1^{delithiation}\right) + \sum_{i=2}^{50}\left(Q_i^{lithiation} - Q_{i-1}^{delithiation}\right)$$

Eq 3c – Li_xSi-trapping: $\sum_{i=2}^{50} Q(Trapped\ LixSi) = \sum_{i=2}^{50}\left(Q_{i-1}^{delithiation} - Q_i^{delithiation}\right)$

2. Explicit expansion for N = 4

Let Q_k ≡ Q_k^lith and D_k ≡ Q_k^delith.

Q(SEI) = (Q1−D1) + (Q2−D1) + (Q3−D2) + (Q4−D3)

Q(trapped LixSi) = (D1−D2) + (D2−D3) + (D3−D4)

3. Cancellation row by row

Row	from Q_SEI	from Q_LixSi
1	Q1 − D1	—
2	Q2 − D1	D1 − D2
3	Q3 − D2	D2 − D3
4	Q4 − D3	D3 − D4

Adding each row:

Row 1 → Q1 − D1

Row 2 → Q2 − D2 (D1 terms cancelled)

Row 3 → Q3 − D3 (Q3 and D2 terms cancel)

Row 4 → Q4 − D4 (Q4 and D3 terms cancel)

Thus, after cancellation we obtain Σ{i=1}^{4}(Q_i − D_i) = Q(tot),irr.

4. General results

Because the same telescoping pattern applies for any N, it follows that

Q_SEI + Q_LixSi = Q_tot,irr……equation 3d $Q\ (tot)_{irr}$

$$= \sum_{i=1}^{50} Q(SEI) \; + \sum_{i=2}^{50} Q(Trapped\ LixSi)$$

for all cycle numbers. Every coulomb of irreversible charge is accounted for exactly once—either in continued SEI growth or in lithium trapped as Li_xSi.

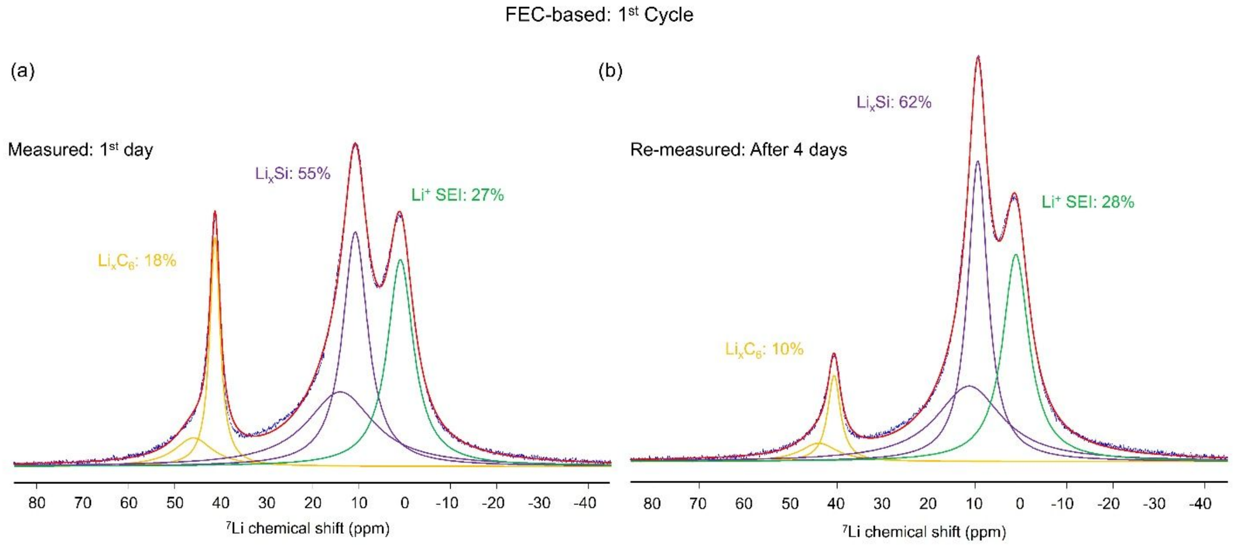

Figure S7. Deconvoluted ^{7}Li NMR spectra of a lithiated silicon–graphite composite anode (first cycle) with the FEC-based electrolyte, measured on day 1 after cell disassembly (a) and re-measured after 4 days (b). Note: These spectra were selected from Figure 8a as a representative example for accurate deconvolution because they are relatively better resolved than the other spectra.
